# Blood Pressure Circadian Variation, Cognition and Brain Imaging in 90+ Year-Olds

**DOI:** 10.3389/fnagi.2019.00054

**Published:** 2019-04-17

**Authors:** Annlia Paganini-Hill, Natalie Bryant, Maria M. Corrada, Dana E. Greenia, Evan Fletcher, Baljeet Singh, David Floriolli, Claudia H. Kawas, Mark J. Fisher

**Affiliations:** ^1^Department of Neurology, School of Medicine, University of California, Irvine, Irvine, CA, United States; ^2^Institute for Memory Impairments and Neurological Disorders, University of California, Irvine, Irvine, CA, United States; ^3^Department of Epidemiology, School of Medicine, University of California, Irvine, Irvine, CA, United States; ^4^Department of Neurology, Center for Neuroscience, University of California, Davis, Davis, CA, United States; ^5^Department of Radiological Sciences, School of Medicince, University of California, Irvine, Irvine, CA, United States; ^6^Department of Neurobiology & Behavior, School of Biological Sciences, University of California, Irvine, Irvine, CA, United States

**Keywords:** ambulatory blood pressure monitoring, cognition, cognitive neurological examination, neuropsychological tests, magnetic resonance imaging

## Abstract

**Purpose**: To analyze the relationship between blood pressure (BP) variables, including circadian pattern, and cognition in 90+ year-olds.

**Methods**: Twenty-four hour ambulatory BP monitoring was completed on 121 participants drawn from a longitudinal study of aging and dementia in the oldest-old. Various measures of BP and its variability, including nocturnal dipping, were calculated. Each person was given both a neuropsychological test battery covering different cognitive domains and a neurological examination to determine cognitive status. Seventy-one participants had a brain magnetic resonance imaging (MRI) scan.

**Results**: Participants ranged in age from 90 to 102 years (mean = 93), about two-thirds were female, and nearly 80% had at least some college education. Mean nocturnal dips differed significantly between cognitively normal (*n* = 97) and impaired individuals (*n* = 24), with cognitively normal participants having on average greater nocturnal dips [6.6% vs. 1.3%, *p* = 0.006 for systolic BP (SBP); 11% vs. 4.4%, *p* = 0.002 for diastolic BP (DBP)]. Nocturnal dips were also related to performance on select cognitive test scores (especially those related to language, recent memory and visual-spatial ability), with individuals who performed below previously established median norms having significantly smaller nocturnal dips (both SBP and DBP) than those above the median. DBP reverse dippers had larger mean white matter hyperintensities (WMH as percent of total brain volume; 1.7% vs. 1.2%, 1.1% and 1.0% in extreme dippers, dippers, non-dippers) and a greater proportion had lobar cerebral microbleeds (CMBs; 44% vs. 0%, 7%, 16%, *p* < 0.05). Impaired participants had higher mean WMH than those with normal cognition (1.6% vs. 1.0% *p* = 0.03) and more tended to have CMB (31% vs. 20%, p = n.s.).

**Conclusion**: These findings suggest that cognitive dysfunction is associated with dysregulation in the normal circadian BP pattern. Further study is warranted of the potential role of WHM and CMB as mediators of this association.

## Introduction

Several studies have found that blood pressure (BP) levels and other BP-related features such as variability, daytime and nighttime measures, and nocturnal dipping are associated with mortality, cardiovascular events, cerebrovascular disease, and subclinical damage to the heart, kidney and vessels (Boggia et al., 2007; Sörös et al., 2013; Diaz et al., 2014; Douma and Gumz, 2018). Although studies show that high BP in middle-age and late-life is associated with increased risk of cognitive decline and dementia (Launer et al., 2000; Kivipelto et al., 2001; Yamada et al., 2003; Whitmer et al., 2005; Arvanitakis et al., 2018), other work suggests that developing hypertension at older ages may protect against dementia (Corrada et al., 2017).

Twenty-four hour ambulatory BP monitoring (ABPM) offers greater precision in estimating average BP than one-time readings and provides information on variability and circadian pattern of BP (O’Brien et al., 2013). While several studies have analyzed the association of BP variability and cognition in the elderly (van Boxtel et al., 1998, 2006; Kanemaru et al., 2001; Axelsson et al., 2008; Nagai et al., 2008; Guo et al., 2010; Conway et al., 2015), none, to our knowledge, have investigated this in the oldest-old (aged 90+ years). Considering the aging of the population with an increasing proportion of those aged 90+ (United Nations Department of Economic and Social Affairs Population Division, 2017) and the increasing risk of cognitive decline and dementia with age (Corrada et al., 2010), elucidating associations with cognitive function, especially of variables which are modifiable, remains vital. Therefore, using data from 24-h ABPM of 90+ year-olds, we analyzed the relationships between various BP measurements, their variability, and circadian pattern of BP and cognition with the aim of finding the variable most closely associated with cognitive function, hypothesizing that BP circadian variation impacts cognitive function.

## Materials and Methods

### Participants

Study subjects were participants in *The 90+ Study*, a study of aging and dementia in individuals aged 90 years and older from the Laguna Woods Village (California) retirement community and its surroundings (Paganini-Hill et al., 2016). Subjects are seen in-person and longitudinally followed every 6 months. At each visit, their medical history is updated and they are given a neuropsychological test battery and a neurological examination as previously described (Whittle et al., 2007). Data from the visit closest in date to the date of ABPM were selected. The study was approved by the Institutional Review Board of the University of California Irvine and 149 participants agreed to and gave written informed consent for 24-h ABPM.

### Ambulatory Blood Pressure Monitoring

Subjects underwent ABPM with a fully automatic device (Sun Tech—Oscar 2 with AccuWi n 3.4.4.0 software). The instrument was worn 24-h (during daytime and sleep) with sampling every 1 h. Systolic BP (SBP) readings greater than 260 mmHg or lower than 70 mmHg as well as diastolic BP (DBP) readings greater than 150 mmHg or lower than 40 mmHg were discarded. Participants were told to follow their normal daily activities during measurements. Daytime measures were considered those between 07:00 and 21:59 and nighttime as those between 22:00 and 06:59.

We represented BP by several indices. Separately for 24-h, daytime and nighttime and for SBP and DBP, we calculated mean, minimum, maximum, standard deviation (SD), coefficient of variation (CV), average real variability (ARV), proportion of high values (SBP ≥140 mmHg, DBP ≥90 mmHg), and proportion of low values (SBP <90 mmHg, DBP <60 mmHg). We also calculated proportion of high BP measurements (either SBP ≥140 mmHg or DBP ≥90 mmHg) and proportion of low BP measurements (either SBP <90 mmHg or DBP <60 mmHg). Separately for SBP and DBP, we calculated nocturnal dip (%) as (1-mean nighttime BP/mean daytime BP) × 100. Subjects were further classified by nocturnal dipping category: extreme dipper (≥20%), dipper (10%–19%), non-dipper (0%–9%), reverse dipper (<0%).

### Neuropsychological Tests

Trained psychometrists administered the neuropsychological test battery. The 10 tests represent different cognitive domains such as language (Verbal Fluency Tests (Animal and Letter F) and Boston Naming Test, BNT), recent memory (California Verbal Learning Tests, CVLT), executive function (Trail Making Tests), visual-spatial ability (Clock Drawing and CERAD Construction), attention/working memory (Digit Span Tests), as well as global cognition [the mini-mental state examination (MMSE) and modified MMSE (3MS)]. Details of the use of these tests and age-adjusted cognitive norms in this population group have been previously presented (Whittle et al., 2007).

### Neurological Examination

Neurological examiners (trained physicians or nurse practitioners) administered the Clinical Dementia Rating scale (Morris, 1993) and assessed functional abilities using the Functional Activities Questionnaire (Pfeffer et al., 1982). Using MMSE and 3MS results and information from their examination they determined the presence or absence of dementia, applying Diagnostic and Statistical Manual of Mental Disorders, 4th edition criteria (American Psychiatric Association, 1994), classifying individuals as normal, cognitively impaired not demented (CIND), or demented.

### Brain Imaging

Some participants underwent brain magnetic resonance imaging (MRI) using a GE Signa HD 3.0 Tesla MRI system as previously described (Bennett et al., 2017). A high-resolution T1-weighted fast-spoiled gradient recalled echo (TR/TE/IT = 7/3/400ms, FOV = 256 × 256 mm, 166 sagittal slices, and 1.0 mm^3^ spatial resolution), fluid attenuation inversion recovery (FLAIR) sequence and susceptibility-weighted imaging (SWI) were acquired. We calculated T1-weighted image tissue volumes using in-house methods described previously (Fletcher et al., 2018). Briefly, brain masks to separate brain from whole head were generated using an atlas-based method (Aljabar et al., 2009) followed by human quality control as needed. Stripped brains were further segmented into four tissue types: gray matter (GM), white matter (WM), cerebrospinal fluid (CSF) and WM hyperintensities (WMH), using a segmentation algorithm designed to enhance accuracy at tissue boundaries (Fletcher et al., 2012) in combination with FLAIR imaging to estimate WMH. Volumes of CSF, GM, WM and WMH were calculated as percent of total brain volume.

MRI were also reviewed by a neuroradiologist (DF), who rated definite cerebral microbleeds (CMBs) presence, number and distribution on SWI using the microbleed anatomical rating scale (MARS; Gregoire et al., 2009). In MARS, definite CMB were defined as small, rounded or circular, well-defined hypodense lesions within brain parenchyma with clear margins ranging from 2 to 10 mm in size on T2*-weighted images, and locations of CMB were classified into deep, lobar, and infratentorial categories.

### Covariates

Age, sex, education, smoking and medical histories of cardiovascular and cerebral vascular diseases [high BP, heart attack, coronary artery disease, congestive heart failure, heart valve disease, stroke, transient ischemic attack (TIA), diabetes] were asked. Consumption of wine, beer and hard liquor (0, <1, 1, 2, 3, 4+ drinks) and participation in physical and other activities (0, 1/4, 1/2, 1, 2, 3–4, 5–6, 7–8, 9+ h) were asked for an average weekday.

### Statistical Analyses

Univariate analyses (calculation of means, standard errors, proportions) were conducted to assess BP metrics and subject characteristics. Mean daytime and nighttime values for each BP metric were compared using paired *t*-tests. To study the association between BP and cognition, we used chi-square and Fisher exact-tests for categorical variables and *t*-tests and analysis of variance (ANOVA) with contrasts for specific group comparisons for continuous variables. All analyses were conducted using SAS statistical software version 9.4 (SAS Institute, Cary, NC, USA). A two-sided *p* < 0.05 was considered statistically significant.

## Results

From the 149 participants agreeing to 24-h ABPM, we eliminated 28 persons who had less than six valid observations during the daytime or the nighttime periods. The characteristics of the remaining 121 subjects with sufficient ABPM readings (ranging from 12 to 24) are shown in Table 1. The age of participants ranged from 90 to 102 (mean ± SE = 93.2 ± 0.3). All participants were Caucasian, except one Asian. Nearly two-thirds (63%) were women and 79% had at least some college education. No subject currently smoked although 46% had smoked in the past. About two-thirds (64%) of participants reported having had high BP. Other cardiovascular and cerebral vascular diseases ranged from 7% for heart attack to 19% for TIA. The number of days between BP monitoring and the cognitive testing visit ranged from 0 to 182 (mean ± SE = 36 ± 2.9). By neurological examination most (80%) participants were classified as normal (*n* = 97), the others as cognitively impaired with CIND (*n* = 21) or dementia (*n* = 3). Normal and cognitively impaired subjects did not differ significantly on baseline characteristics except time spent in other indoor activities (85% vs. 54% for 2 + h/day, *p* = 0.004; Table 1). Table 2 shows the BP metrics for 24-h, daytime and nighttime periods for the 121 subjects. Paired *t*-tests verified significant differences between daytime and nighttime BP variables signifying a circadian pattern.

**Table 1:** Characteristics of 121 subjects with 24-h ambulatory blood pressure monitoring (ABPM).

	All subjects (*n* = 121)	Normal (*n* = 97)	Cognitively impaired (*n* = 24)
	Mean ± SE	Mean ± SE	Mean ± SE
*Age (years)*	93.2 ± 0.3	93.4 ± 0.3	92.8 ± 0.5
*Days between BP and cognitive*	36 ± 2.9	35 ± 3.3	40 ± 5.9
*testing*
	***N*** (%)	***N*** (%)	***N*** (%)
*Female*	76 (63%)	58 (60%)	18 (75%)
*Education*			
<College	25 (21%)	19 (20%)	6 (25%)
Some college, college graduate	57 (47%)	47 (48%)	10 (42%)
Post-graduate	39 (32%)	31 (32%)	8 (33%)
*Past smoker*	56 (46%)	49 (51%)	7 (29%)
*Alcohol intake drinks/day*			
Wine—none	58 (48%)	46 (47%)	12 (50%)
Beer—none	91 (75%)	70 (72%)	21 (88%)
Hard liquor—none	84 (69%)	67 (69%)	17 (71%)
*Activities* (missing 3 active, 4 other in normal subjects)			
Active outdoor ≥ 15 min/day	34 (29%)	29 (31%)	5 (21%)
Active indoor ≥ 30 min/day	61 (52%)	46 (49%)	15 (63%)
Other outdoor ≥ 15 min/day	33 (28%)	26 (28%)	7 (29%)
Other indoor ≥ 2 h/day	92 (79%)	79 (85%)	13 (54%)*
*Cardiovascular and cerebral vascular disease history*			
High blood pressure	77 (64%)	63 (65%)	14 (58%)
Heart attack	9 (7%)	7 (7%)	2 (8%)
Coronary artery disease	19 (16%)	13 (13%)	6 (25%)
Congestive heart failure	10 (8%)	8 (8%)	2 (8%)
Heart valve disease	11 (9%)	10 (10%)	1 (4%)
Stroke	11 (9%)	9 (9%)	2 (8%)
TIA	23 (19%)	20 (21%)	3 (12%)
Diabetes	14 (12%)	11 (11%)	3 (12%)

*p = 0.004 for difference between normal and cognitively impaired subjects by chi-square test.

**Table 2:** Twenty-four hour BP levels and variability measurements in 121 subjects.

	24-h	Daytime 07:00–21:59	Nighttime 22:00–06:59
	Mean ± SE	Mean ± SE	Mean ± SE
*Systolic Blood Pressure*			
Mean	137 ± 1.3	141 ± 1.4	132 ± 1.5
Minimum	108 ± 1.4	115 ± 1.4	113 ± 1.6
Maximum	169 ± 1.6	168 ± 1.6	154 ± 1.7
Standard deviation	16.7 ± 0.40	16.4 ± 0.43	13.8 ± 0.47
Coefficient of variation	0.12 ± 0.003	0.12 ± 0.003	0.11 ± 0.004
ARV	16.1 ± 0.41	17.2 ± 0.52	15.1 ± 0.60
% high (≥140 mmHg)	44.8 ± 2.5	50.8 ± 2.6	35.3 ± 2.9
% low (<90 mmHg)	1.0 ± 0.3	0.5 ± 0.2	1.8 ± 0.6
Nocturnal dip (%)	5.5 ± 0.8		
*Diastolic Blood Pressure*			
Mean	67 ± 0.8	70 ± 0.9	63 ± 0.9
Minimum	49 ± 0.7	54 ± 0.8	51 ± 0.9
Maximum	92 ± 1.3	91 ± 1.3	77 ± 1.3
Standard deviation	11.2 ± 0.30	11 ± 0.33	8.7 ± 0.36
Coefficient of variation	0.17 ± 0.004	0.16 ± 0.004	0.14 ± 0.006
ARV	10.2 ± 0.28	11 ± 0.36	9.1 ± 0.41
% high (≥90 mmHg)	7.3 ± 1.1	10 ± 1.5	3.0 ± 0.8
% low (<60 mmHg)	31 ± 2.3	24 ± 2.2	42 ± 3.0
Nocturnal dip (%)	9.9 ± 0.9		
*Blood Pressure*			
% high (SBP≥140 or DBP ≥90)	45 ± 2.5	52 ± 2.6	36 ± 2.9
% low (SBP<90 or DBP<60)	31 ± 2.3	24 ± 2.2	42 ± 3.0

*All paired t-tests comparing daytime and nighttime measurements are statistically significant with *p* < 0.0001 except Systolic BP (SBP) average real variability (ARV; *p* = 0.003), SBP % low (*p* < 0.05), diastolic BP (DBP) minimum (*p* = 0.0003), SBP minimum (*p* = 0.20)*.

### Nocturnal Dips and Association With Cognitive Status

Normal individuals did not differ from cognitively impaired individuals on most BP metrics. Impaired persons had lower means of daytime SBP, % high SBP (≥140 mmHg) and % high BP (SBP ≥140 or DBP ≥90; Table 3). The most significant differences between the two groups were mean nocturnal dips with cognitively normal participants having on average greater nocturnal dips (6.6% vs. 1.3%, *p* = 0.006 for SBP; 11% vs. 4.4%, *p* = 0.002 for DBP). Likewise, cognitively normal individuals were more likely than impaired individuals to be nocturnal dippers (37% vs. 8% for SBP; 54% vs. 33% for DBP) while impaired individuals were more likely to be reverse dippers (42% vs. 21% for SBP, *p* = 0.03; 33% vs. 10% for DBP, *p* = 0.01; Table 4).

**Table 3:** Daytime BP levels and variability measurements by cognitive status.

	Normal (*n* = 97)	Cognitively impaired (*n* = 24)
	Mean ± SE	Mean ± SE
*Systolic Blood Pressure*		
Mean	142 ± 1.5	135 ± 3.0*
Minimum	116 ± 1.6	110 ± 3.1
Maximum	169 ± 1.9	164 ± 3.3
Standard deviation	16.1 ± 0.46	17.3 ± 1.08
Coefficient of variation	0.12 ± 0.003	0.12 ± 0.007
ARV	17.0 ± 0.57	18.1. ± 1.2
% high (≥140 mmHg)	53 ± 2.9	40 ± 5.7*
% low (<90 mmHg)	0.4 ± 0.16	1.3 ± 0.74
Nocturnal dip (%)	6.6 ± 0.8	1.3 ± 1.7*
*Diastolic Blood Pressure*		
Mean	71 ± 0.99	68 ± 2.0
Minimum	55 ± 0.92	51 ± 1.5
Maximum	92 ± 1.4	87 ± 3.2
Standard deviation	10.8 ± 0.35	11.2 ± 0.88
Coefficient of variation	0.16 ± 0.004	0.16 ± 0.007
ARV	11.1 ± 0.40	11.6 ± 0.90
% high (≥90 mmHg)	11 ± 1.7	8.1 ± 3.2
% low (<60 mmHg)	22 ± 2.4	30 ± 5.2
Nocturnal dip (%)	11 ± 1.0	4.4 ± 2.1*
*Blood Pressure*		
% high (SBP ≥140 or DBP ≥90)	54 ± 2.9	41 ± 5.6*
% low (SBP<90 or DBP<60)	22 ± 2.4	30 ± 5.2

*Statistically significant (p < 0.05) difference between normal and cognitively impaired subjects by t-test; *p* = 0.006 for SBP nocturnal dip and *p* = 0.002 for DBP nocturnal dip.

**Table 4:** Nocturnal dipping by cognitive status.

	Normal (*n* = 97)	Cognitively impaired (*n* = 24)	*p*-value for difference
*SBP Nocturnal Dip (%)*			
Mean ± SE	6.6 ± 0.8	1.3 ± 1.7	0.006
Dip category			0.02
Extreme dipper (≥20%)	7 (7%)	1 (4%)	
Dipper (10–19%)	29 (30%)	1 (4%)	
Non-dipper (0–9%)	41 (42%)	12 (50%)	
Reverse dipper (<0%)	20 (21%)	10 (42%)	
*DBP Nocturnal Dip (%)*			
Mean ± SE	11 ± 0.96	4.4 ± 2.1	0.002
Dip category			0.008
Extreme dipper (≥20%)	18 (19%)	0 (0%)	
Dipper (10–19%)	34 (35%)	8 (33%)	
Non-dipper (0–9%)	35 (36%)	8 (33%)	
Reverse dipper (<0%)	10 (10%)	8 (33%)	

*p-value for difference in means by t-test and for difference in proportions by Fisher exact test*.

### Nocturnal Dips and Association With Cognitive Measures

Over three-quarters of participants surpassed the median norm previously established for their age group on most cognitive tests. Performance was poorer on tests of executive function or attention/working memory (60%–70% above median norm; data not shown). Nocturnal dips were related to performance on cognitive test scores related to language (BNT), recent memory (CVLT), and visual-spatial ability (CERAD Construction; Table 5). Individuals who performed below the median norm had significantly smaller nocturnal dips (both SBP and DBP) than those above the median. Table 6 cross-classifies individuals on these same cognitive tests as below/above the median norm and the four dipping categories. Similar results were observed; reverse dippers performed the worst on these three tests. Cognitively impaired individuals by neurological examination were also more likely to be reverse dippers.

**Table 5:** Nocturnal dips (Mean ± SE) by cognitive tests scores (below and above median).

		Sample size	Mean ± SE of nocturnal dip	*p*-value for difference
*SBP Nocturnal Dip*				
Boston Naming Test	<median	24	2.2 ± 1.7	0.007
	>median	80	7.3 ± 0.88	
CVLT Cued Delay	<median	19	1.5 ± 1.9	0.01
	>median	99	6.7 ± 0.82	
CERAD Construction	<median	21	1.8 ± 1.8	0.02
	>median	85	6.6 ± 0.89	
*DBP Nocturnal Dip*				
Boston Naming Test	<median	24	5.8 ± 2.3	0.006
	>median	80	11.9 ± 0.97	
CVLT Cued Delay	<median	19	6.2 ± 2.6	0.06
	>median	99	10.9 ± 0.96	
CERAD Construction	<median	21	4.8 ± 2.0	0.005
	>median	85	11.5 ± 1.0	

*p-value for difference in means by t-test*.

**Table 6:** Percent normal (above median norm) on cognitive tests and on neurological examination by nocturnal BP dipping category.

	Extreme dippers	Dippers	Non-dippers	Reverse dippers	*p*-value 4 groups	*p*-value Reverse dippers vs. other
*SBP Nocturnal Dip*	*n* = 8	*n* = 30	*n* = 53	*n* = 30		
Boston Naming Test	100%	85%	77%	61%	0.10	0.05
CVLT Cued Delay	88%	97%	83%	70%	0.04	0.04
CERAD Construction	88%	96%	73%	75%	0.08	0.56
Neurological examination	88%	97%	77%	67%	0.07	0.06
*DBP Nocturnal Dip*	*n* = 18	*n* = 42	*n* = 43	*n* = 18		
Boston Naming Test	87%	79%	84%	38%	0.01	0.002
CVLT Cued Delay	94%	83%	85%	71%	0.31	0.15
CERAD Construction	94%	89%	71%	67%	0.06	0.17
Neurological examination	100%	81%	81%	56%	0.008	0.009

*p-value for difference in proportions by chi-square or Fisher exact test*.

### Nocturnal Dips and Association With Structural Brain Measures

Brain MRI was available for 71 participants. Reasons for missing MRI data were bodily presence of hardware (e.g., pacemaker, metal implant; *n* = 21), other condition (e.g., too weak, could not tolerate time in scanner; *n* = 4), refused (*n* = 1), bad imaging (*n* = 1), not part of imaging study (*n* = 23).

Both mean WMH volume and presence of CMB were related to DBP nocturnal dip and to cognitive status (Table 7). Mean WMH was highest in DBP reverse dippers (1.7% vs. 1.2%, 1.1% and 1.0% in extreme dippers, dippers, non-dippers) and was significantly different between reverse dippers and non-dippers (*p* < 0.05). Mean WMH was also higher in those with impaired cognition (1.6%, vs. 1.0% *p* = 0.03). Impaired participants were also more likely than those with normal cognition to have CMB (31% vs. 20%), although the difference was not significant. A greater proportion of DBP reverse dippers had lobar CMB than the others (44% vs. 0%, 7%, 16% in extreme dippers, dippers, non-dippers, *p* < 0.05). In contrast, a greater proportion of DBP extreme dippers had deep CMB than the others (30% vs. 4%, 0%, 0% in dippers, non-dippers, reverse dippers, *p* < 0.05).

**Table 7:** Brain magnetic resonance imaging (MRI) findings by nocturnal BP dipping category and cognitive status.

	Dipping category				Cognitive status	
	Extreme dippers	Dippers	Non-dippers	Reverse dippers	Normal	Impaired
**% of total brain volume (mean ± SE)**						
*SBP Nocturnal Dip*	*n* = 5	*n* = 18	*n* = 34	*n* = 13	*n* = 54	*n* = 16
Cerebral spinal fluid	27.7 ± 0.93	28.3 ± 0.38	28.4 ± 0.35	28.4 ± 0.56	28.2 ± 0.25	28.7 ± 0.53
Gray matter	41.7 ± 0.59	41.0 ± 0.38	41.0 ± 0.26	41.6 ± 0.51	41.3 ± 0.22	40.9 ± 0.39
White matter	29.0 ± 0.94	29.7 ± 0.36	29.5 ± 0.39	28.6 ± 0.52	29.5 ± 0.27	28.9 ± 0.55
White matter hyperintensities	1.5 ± 0.52	0.99 ± 0.17	1.1 ± 0.12	1.4 ± 0.31	1.0 ± 0.11	1.6 ± 0.96*
*DBP Nocturnal Dip*	*n* = 10	*n* = 26	*n* = 25	*n* = 9		
Cerebral spinal fluid	28.6 ± 0.58	28.4 ± 0.41	28.0 ± 0.32	28.7 ± 0.81		
Gray matter	41.3 ± 0.34	40.9 ± 0.25	41.4 ± 0.38	41.2 ± 0.65		
White matter	29.0 ± 0.57	29.5 ± 0.38	29.7 ± 0.42	28.5 ± 0.79		
White matter hyperintensities	1.2 ± 0.35	1.1 ± 0.15	1.0 ± 0.13	1.7 ± 0.39		
**Cerebral microbleeds (proportion with CMB)**						
*SBP Nocturnal Dip*	*n* = 5	*n* = 18	*n* = 35	*n* = 13		
Any CMB	1 (20%)	3 (17%)	9 (26%)	3 (23%)	11 (20%)	5 (31%)
Infratentorial CMB	0 (0%)	1 (6%)	2 (6%)	0 (0%)	2 (3%)	1 (6%)
Deep CMB	1 (20%)	2 (11%)	1 (3%)	0 (0%)	4 (7%)	0 (0%)
Lobar CMB	0 (0%)	1 (6%)	5 (17%)	3 (23%)	6 (11%)	4 (25%)
*DBP Nocturnal Dip*	*n* = 10	*n* = 27	*n* = 25	*n* = 9		
Any CMB	3 (30%)	3 (11%)	6 (24%)	4 (44%)		
Infratentorial CMB	1 (10%)	0 (0%)	2 (8%)	0 (0%)		
Deep CMB	3 (30%)	1 (4%)	0 (0%)	0 (0%)*		
Lobar CMB	0 (0%)	2 (7%)	4 (16%)	4 (44%)*		

*p < 0.05 for difference in means between normal and impaired subjects by t-test or for difference in proportions among the four dipping categories by Fisher exact test.

## Discussion

To our knowledge, our study is the first to evaluate 24-h BP measures, including circadian BP pattern, and their association with cognition in individuals aged 90 years and older. In this oldest-old sample, mean nocturnal dip (% difference in mean daytime and mean nighttime BP measures) differed between cognitively normal and impaired individuals for both SBP and DBP. Classification of individuals by dipping categories showed that impaired individuals compared with cognitively normal individuals were less likely to be dippers (SBP 8% vs. 37%, DBP 33% vs. 54%) and more likely to be reverse dippers (SBP 42% vs. 21%, DBP 33% vs. 10%).

Studies examining nocturnal dipping and cognition in the elderly are limited and those using community-dwelling samples even more so. Three studies included only hypertensive patients (Kanemaru et al., 2001; van Boxtel et al., 2006; Nagai et al., 2008). Classifying 88 Japanese patients from a hypertension clinic (mean age = 71, range = 37–90) into three cognitive groups, Kanemaru et al. (2001) found the severely affected group had smaller differences of day-night BP (i.e., less nocturnal dipping), which was significant for DBP. In another Japanese study, Nagai et al. reported a positive association between nocturnal SBP dipping and MMSE score in 55 untreated hospitalized hypertensives (aged ≥ 70, mean = 73; Nagai et al., 2008). However, an investigation of 86 patients aged 40–80 years from a hypertension clinic in the Netherlands reported no association between nocturnal dipping and cognitive measures (van Boxtel et al., 2006). Two additional non-community based studies showed a positive association between nocturnal dipping and cognitive function based on MMSE; the first utilized 99 nursing home/geriatric hospital subjects (mean age 80 years; Ohya et al., 2001) and the second 224 patients hospitalized for lacunar infarctions (Yamamoto et al., 2011). In a sample of 115 community residents aged 28–82 years from the Maastricht Aging Study, non-dippers showed lower levels of both memory and sensorimotor speed scores, but extreme dippers were not analyzed separately (van Boxtel et al., 1998). In a study of 79 81-year-old men in a Swedish community, nocturnal SBP dipping was associated with lower cognitive performance (Axelsson et al., 2008). In a study of 144 Japanese community dwelling persons (aged ≥ 50 years, mean = 68), MCI had a J-shaped relationship with dipping category with frequency of MCI being about 32%, 13%, 30%, and 50% (from extreme dipper to reverse dipper) subjects (Guo et al., 2010). In our study of the oldest-old, we also found that reverse dippers had the highest frequency of cognitive impairment (SBP 12%, 3%, 23%, and 33%; DBP 0%, 19%, 17%, 44%). Another community-based study of 319 older (≥60 years, mean = 72) adults from the San Diego Population Study found greater nighttime SBP dipping significantly associated in a linear manner across quartile groups (cutoffs not given) with better cognitive function (Montreal Cognitive Assessment; Conway et al., 2015). Greater nighttime dipping was modestly associated with better performance on the immediate recall portion of the Hopkins Verbal Learning Test–Revised, which measures memory, and on the Trail-Making Test Part A, which measures a number of domains including cognitive flexibility, visuospatial processing, and executive function. We also found that mean nocturnal dips differed between participants above and below the median performance on select cognitive tests including BNT, CVLT cued delay and CERAD construction, tests related to the domains of naming, recent memory and construction. Dippers were also more likely than non-dippers to perform better on these three cognitive tests. Although we found no association with global measures of cognition, previous studies have underscored the necessity of more robust assessment of memory function—especially delayed recall—in cognitive screening (Lacy et al., 2015).

These findings suggest that cognitive dysfunction may be associated with dysregulation in BP pattern. If confirmed, and if nocturnal dipping patterns are shown to precede cognitive impairment in longitudinal studies, the addition of ABPM to clinic-based BP may allow identification of older persons at higher risk for cognitive impairment beyond other known risk factors. However, analyses of future BP intervention studies will need to examine whether manipulation of dipping pattern would improve the trajectory of cognitive function in older persons.

Although current guidelines recommend the use of ABPM for diagnosis of hypertension, its role as a guide to prescribing the appropriate drug and dosage over time is lacking (O’Brien and Dolan, 2016). Studies in recent years on the use of chronotherapy in hypertension suggest that nighttime administration of antihypertensives improves the 24-h BP profiles of patients (Smolensky et al., 2016; Bowles et al., 2018). However, only the American Diabetic Association has made such a recommendation (American Diabetes Association, 2014).

Whether these events are regulated by a common central mechanism of BP variability needs further study. Dipping patterns can be explained by physiological changes in circadian rhythm, though its underlying mechanisms are not fully understood. Suggested factors include sympathetic nervous system overactivation (perhaps due to obesity, diabetes, metabolic syndrome), obstructive sleep apnea, dysfunction in activity of renin-angiotensin-aldosterone axis, sodium intake, and chronic kidney disease (Dubielski et al., 2016; Douma and Gumz, 2018). Some studies have found associations with cerebral WM lesions, as we did (Sander et al., 2000), asymptomatic lacunar infarction (Kario et al., 1996), reduced total brain matter (Nagai et al., 2008), and small vessel disease (Yamaguchi et al., 2014). Both ischemic (microinfarctions) and hemorrhagic (CMB) small vessel disease have been linked to dysfunctional cerebral blood flow as a consequence of altered BP regulation (Fisher, 2016). We also found that lobar microbleeds were associated with reverse dipping, while deep microbleeds were associated with extreme dipping. These intriguing findings, which raise the possibility of different hemodynamic mechanisms for these two kinds of microbleeds, warrant further study. Note that lobar and deep microbleeds are thought to be driven by cerebral amyloid angiopathy and hypertension, respectively (Greenberg et al., 2009). Overall, these results suggest that cerebrovascular disease may mediate the link between cognitive dysfunction and BP dysregulation.

Several strengths and limitations of this study warrant mention. Our major strength is the well-characterized participants who are part of a longitudinal epidemiologic study of aging and dementia. These community-dwelling older adults include women and men as well as hypertensives and non-hypertensives. Additionally, we used a neuropsychological test battery covering numerous cognitive domains and a structured neurological examination. One limitation is that ABPM, although noninvasive, was unsuccessful in nearly 20% of subjects; some participants found the cuff inflation or sleep disturbance intolerable. Additionally, we used the fixed-time method (hourly) of measuring ABPM, which would not detect differences in short-term BP variability. Also, we selected daytime and nighttime measures by time of day and not by sleep/wake pattern. Nonetheless, ABPM provides information on BP variability, the day and night BP profile, and nocturnal dipping pattern not available from one-time clinical measurement (O’Brien et al., 2013). Our sample was recruited from a highly educated, moderately affluent population, which may limit the generalizability of our findings to populations with less education. Our cross-sectional design does not allow us to determine the temporal direction of the associations or causal inferences. Analysis of MRI imaging as well as longitudinal follow-up for cognitive decline in these participants may help answer remaining questions.

In conclusion, we observed associations of nocturnal BP dipping with cognitive status, performance on select cognitive tests and MRI evidence of cerebral microvascular disease in a group of apparently healthy oldest-old individuals. This suggests that cognitive dysfunction is associated with dysregulation in the normal circadian BP pattern and that microvascular disease may play a role as a mediator of this phenomenon. Further studies incorporating longitudinal observations with brain imaging are warranted to fully evaluate these findings.

## Author Contributions

AP-H, MC, CK and MF made substantial contributions to the conception or design of the work. NB, DG, EF, BS and DF contributed to the acquisition of the data. AP-H and NB contributed to the analysis. AP-H and MF contributed to the interpretation of data. AP-H and MF drafted the work and the other authors revised it critically for important intellectual content. All authors approved the final version to be published and agreed to be accountable for all aspects of their work in ensuring that questions related to the accuracy or integrity of any part of the work are appropriately investigated and resolved.

## Conflict of Interest Statement

The authors declare that the research was conducted in the absence of any commercial or financial relationships that could be construed as a potential conflict of interest.
